# Physical Therapy Rehabilitation Facilitated With Mindfulness Training for a Post-surgical Tibial Fracture Managed With Ilizarov: A Case Report

**DOI:** 10.7759/cureus.114388

**Published:** 2026-08-11

**Authors:** Sagar Rathod, Siddhi Patrekar, Sandeep Shinde, Krutika Dhawde

**Affiliations:** 1 Department of Musculoskeletal Sciences, Krishna College of Physiotherapy, Krishna Vishwa Vidyapeeth (Deemed To Be University), Karad, IND; 2 Department of Pediatric Neonatal Sciences, Krishna Vishwa Vidyapeeth (Deemed To Be University), Karad, IND

**Keywords:** ilizarov, mindfulness, physical therapy, post-surgical recovery, rehabilitation, tibial fracture

## Abstract

Tibial fractures occur after low- and high-energy trauma caused by indirect and direct impacts, respectively. Surgical site infection (SSI) after open reduction and internal fixation has significant socioeconomic and quality-of-life effects for the patient. Tibia fractures are a common and often complicated injury that can lead to infection after surgical fixation. Corrective surgery requires an external fixator, such as the Ilizarov fixator, which helps in non-union fractures to reunite. After the external fixation, the patient is immobilised for a long period of time. Therefore, physiotherapy management helps prevent postoperative complications occurring due to prolonged immobilisation, which helps achieve functional independence and facilitates the patient to come to a near-normal life as soon as possible. In young age fracture cases because of immobility, there is an immense level of stress and anxiety, which affects the patient's well-being and quality of life. Thus, mindfulness therapy, along with the structured physiotherapy protocol, helps the patient to reduce stress and anxiety and improve sleep quality and quality of life. This case highlights that early, individualized postoperative physiotherapy combined with mindfulness therapy plays a vital role in managing patients with infected proximal tibial fractures treated with Ilizarov fixation. Addressing both physical impairments and psychological well-being through a multidisciplinary rehabilitation approach can accelerate functional recovery, reduce complications associated with prolonged immobilization, improve quality of life, and facilitate an earlier return to independent daily activities*.*

## Introduction

Fractures that are suspected of being proximal tibia fractures that extend into the proximal metaphyseal region of the bone. Fractures that extend into the proximal metaphyseal region of the bone are suggestive of proximal tibia fractures [1]. The large metaphyseal area on the proximal tibia narrows distally to form a triangle. The long tibia shaft articulates with the distal femur, talus, and fibula [2]. A tibia fracture can take many different forms and happen at any age. Simple fractures with minimal soft-tissue injuries can be treated with a brace or plaster cast; more complex fractures require reconstructive surgery, osteosynthesis, or even amputation [3]. Open fractures are frequently associated with high-energy trauma and represent a higher risk of infection due to the surrounding soft-tissue damage and the risk of environmental pollutants entering the fracture site and communicating with it [4]. Kim et al. classified the high-energy open fractures into A, B, and C groups according to the Gustilo-Anderson system based on the extent of soft tissue destruction, the need for vascular repair, and the poor prognosis [4]. Type 3B describes open fractures with periosteal stripping, bone exposure, and significant soft tissue damage; type 3A describes high-energy trauma, regardless of the size of the incision, or open fractures with adequate soft tissue coverage of a broken bone despite severe soft tissue laceration or flaps [5]. The Ilizarov technique is primarily utilized in severe type III-B and type III-C open fractures, where the risk of deep infection prevents the safe use of standard internal plates or rods. Additionally, this ring-fixator method is the gold standard for treating infected non-unions (where a bone fails to heal after previous surgeries). The tibia is prone to fractures due to its exposed position, making it a common injury target. Tibia fractures occur at a rate of 8.1-37.0 per 100,000 individuals [6]. Professor Gavriil Abramovich Ilizarov (1921-1992) of Russia developed a novel form of circular/ring external fixator in the 1950s. Ring external fixators are used to treat difficult cases of infection non-union, limb shortening by bone lengthening, and deformity repair in joints and bones, either alone or in combination [7]. Ring fixators work on four essential principles: distraction neo-osteogenesis, compression at the delayed or non-union site, acute compression, and bone lengthening [8].

Postoperative physiotherapy focuses on early mobilization through gentle range-of-motion exercises for adjacent joints, isometric muscle strengthening, and progressive weight-bearing. Additionally, it incorporates pinning-site care education, edema control, and gait training with assistive devices to optimize mechanical stability and prevent contractures. Patients are usually anxious before and after surgery. Mindfulness-based therapy helps the patient to recover from post-operative pain and anxiety and promotes emotional well-being [9]. Mindfulness is a condition of deep sensory awareness in the present moment, revealing, responsive, and self-referential judgment of the inner experience [10]. Mindfulness practice allows the patient to truly experience what is happening in the present moment by paying attention to and becoming aware of their emotional experiences, which generally occurs postoperatively [11]. Mindfulness can be a useful tool in injury-rehabilitation programs because it can help people relax both mentally and physically, communicate with their physical and mental state, and accept their status as an injured person. This allows for a more effective focus on rehabilitation [12].

Mindfulness therapy is a psychological intervention that involves intentionally focusing attention on the present moment with an attitude of openness and non-judgmental awareness. It has been shown to reduce stress, anxiety, depression, pain perception, and sleep disturbances while improving emotional regulation and quality of life. In rehabilitation settings, mindfulness-based interventions have been increasingly incorporated as adjuncts to conventional physiotherapy to improve psychological resilience, treatment adherence, and overall recovery in patients with chronic pain and musculoskeletal disorders.

The present case is unique because it describes the rehabilitation of an eight-month-old infected proximal tibial fracture managed with an Ilizarov external fixator using a combined approach of structured physiotherapy and mindfulness therapy. Although previous studies have focused primarily on fracture union and surgical outcomes, this case emphasizes holistic rehabilitation addressing both physical impairments and psychological well-being. To the best of our knowledge, limited literature has reported the integration of mindfulness therapy with structured physiotherapy in patients undergoing Ilizarov fixation for infected tibial fractures. The patient primarily presented with pain, lower-limb stiffness, muscle weakness, impaired gait and functional mobility, prolonged immobilization following Ilizarov fixation, and associated psychological distress, including stress, anxiety, and poor sleep quality.

This case report explores the effect of mindfulness therapy along with the physical rehabilitation in a patient with post-proximal tibial fracture managed with Ilizarov. The goal is to look at how this integrative approach might improve the patient's general health, pain management, and physical recovery.

## Case presentation

A 27-year-old male patient complaining of a restricted range of the right leg, unable to bear weight on the right leg, unable to climb stairs, and reduced functional performance of the affected limb in activities of daily living. The patient's height was 173 cm, weight was 70 kg, and body mass index (BMI) was 23.4 kg/m^2^.

According to the patient's history, in 2023, he was admitted to a tertiary care hospital after a road traffic accident. He was diagnosed with a type III-B open fracture and underwent open reduction and internal fixation (ORIF) with plate osteosynthesis and extracutaneous plate application to the right leg, along with wound debridement. Radiological investigations revealed a fracture of the middle third of the right tibial shaft. Limb length discrepancy developed after surgery. After surgery, he experienced difficulty with prolonged standing, stair climbing, and long-distance walking. Eight months later, he noticed a wound on his right leg, which initially measured approximately 1 × 1 cm and subsequently progressed to 12 × 8 cm. Radiological investigations were repeated and revealed non-union of the middle third of the right tibial shaft fracture. On Day 1, the patient underwent application of an Ilizarov ring fixator for the non-union of the middle-third tibial shaft fracture.

The patient was a young adult male with no known history of diabetes mellitus, hypertension, tuberculosis, osteoporosis, or other chronic systemic illnesses. No history of smoking, alcohol abuse, or other significant comorbidities could adversely affect fracture healing. The patient had no known drug allergies. Family history was non-contributory, with no known hereditary musculoskeletal disorders, metabolic bone diseases, or genetic conditions associated with impaired bone healing. Following the prolonged treatment course and immobilization, the patient experienced psychological distress characterized by increased stress, anxiety regarding recovery and return to daily activities, disturbed sleep, and reduced quality of life. These psychosocial factors were considered during rehabilitation, and mindfulness therapy was incorporated alongside structured physiotherapy to address psychological well-being.

The patient sustained an infected proximal tibial fracture approximately eight months before presentation. Initial management consisted of surgical fixation; however, postoperative surgical site infection resulted in delayed fracture healing/non-union. Revision surgery was subsequently performed using an Ilizarov external fixator to provide stable fixation, facilitate infection control, and promote fracture union. Despite satisfactory surgical stabilization, the patient continued to experience pain, joint stiffness, lower-limb muscle weakness, impaired gait, and reduced functional independence because of prolonged immobilization. These persistent impairments necessitated comprehensive physiotherapy rehabilitation combined with mindfulness therapy.

Clinical examination revealed pain around the proximal tibia and pin sites, restricted knee and ankle range of motion secondary to prolonged immobilization, reduced quadriceps and lower-limb muscle strength, impaired weight-bearing ability, altered gait pattern, and decreased functional independence in activities of daily living. The patient also reported significant psychological distress, including stress, anxiety, poor sleep quality, and reduced quality of life during the prolonged treatment period.

The patient underwent structured progressive physiotherapy intervention for one year, along with mindfulness therapy for six sessions per week, lasting 40-60 minutes, focusing mainly on the restricted range of motion, pain reduction, functional mobility, and postoperative anxiety. Progressive improvement was noted in every outcome of the patient, along with calmness of mind.

**Figure 1:**
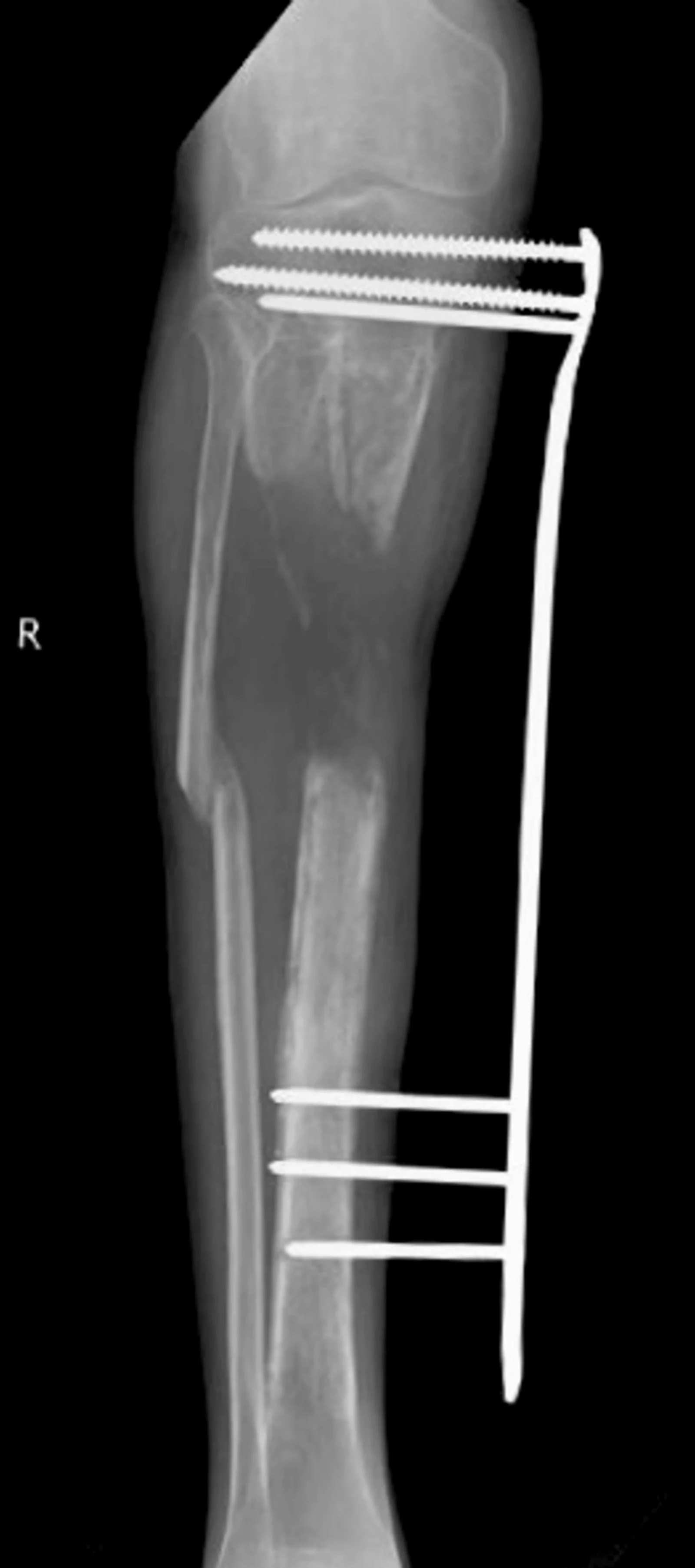
Pre-operative X-ray of the right leg

Preoperative X-ray revealed nonunion of a proximal one-third tibial shaft fracture with an associated fibular shaft fracture in the right leg. Previous internal fixation with plate osteosynthesis and screws is evident. Persistent fracture line and absence of complete cortical bridging are suggestive of established non-union, as shown in Figure 1.

**Figure 2:**
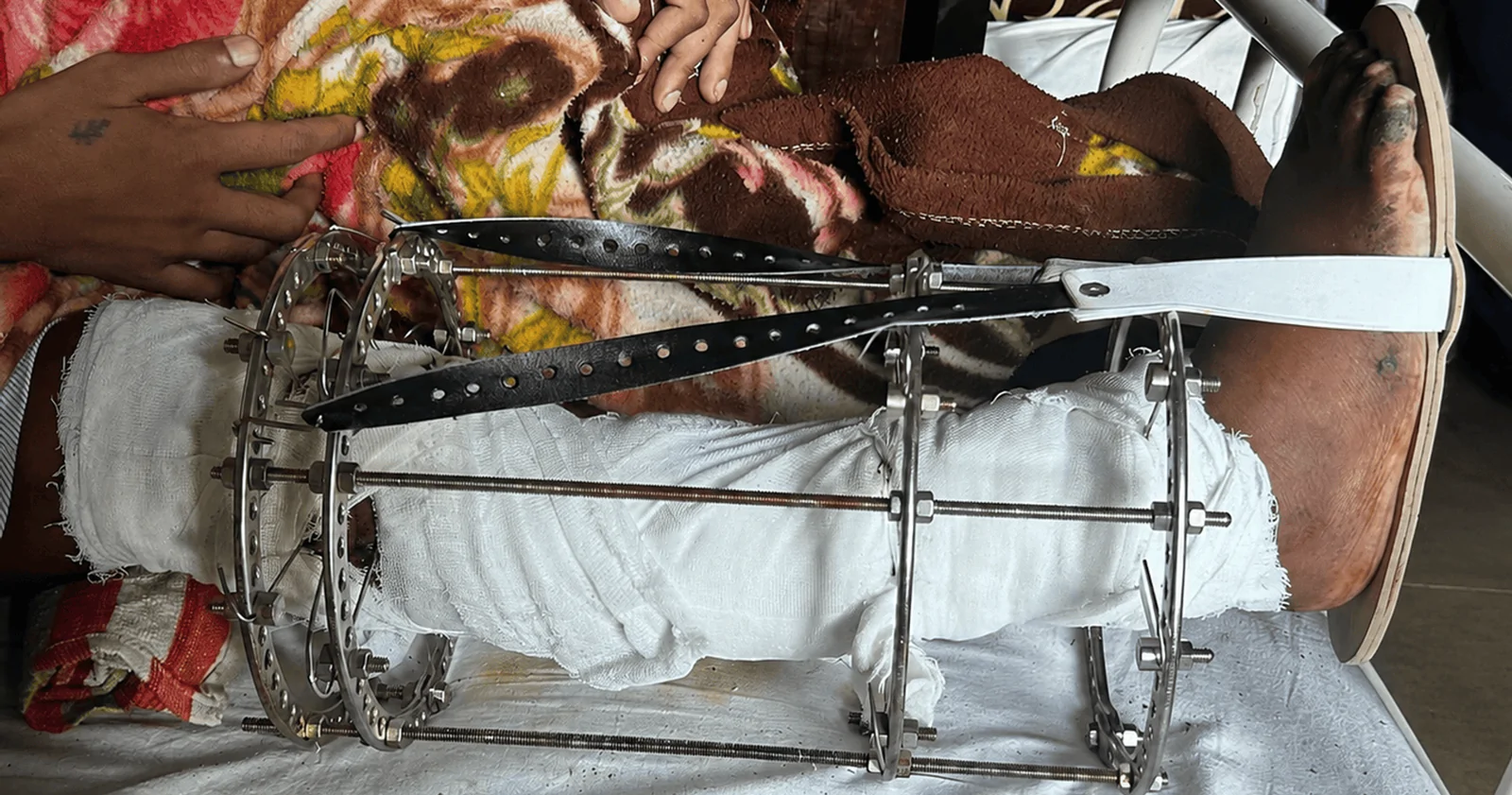
Post Ilizarov fixator application of the right leg

Post Ilizarov fixator application showed correction of the limb length discrepancy. Post fixation, there was an appearance of swelling, as shown in Figure 2.

The patient was educated about his condition and was informed about the findings, physical assessment results, and recommended course of therapy, and informed verbal consent was taken. After detailed examination, the physiotherapy protocol was planned according to the needs of the patient, which will make him functionally independent as soon as possible. Table 1 presents the detailed physiotherapy management along with this mindfulness-based stress reduction therapy, which was administered to the patient to reduce postoperative stress and anxiety, which is significantly seen in young age traumatic fracture patients. The procedure of the mindfulness therapy was explained to the patient. Mindfulness therapy, which includes meditation along with the guided imagination, was given to patients along with the physiotherapy protocol once a day for 30 minutes to help them feel better and reduce anxiety and stress postoperatively.

**Table 1:** Physiotherapy intervention ROM: range of motion

Month	Physiotherapy intervention	Frequency
Month 1	Cryotherapy (10mins), ankle-toe movements, static quadriceps, hamstrings, and gluteal exercises, assisted straight leg raise, passive-assisted knee ROM, heel slides, calf stretching, bed mobility training, bedside sitting balance, non-weight-bearing walker ambulation	10 reps × 3 sets
Month 2	Active-assisted knee ROM, active ankle ROM, straight leg raising in all directions, dynamic quadriceps exercises, hamstring strengthening, hip abduction and extension exercises, seated knee extension, transfer training, walker-assisted ambulation	10 reps × 3 sets
Month 3	Active ROM exercises, theraband strengthening for hip and knee, seated marching, sit-to-stand training, partial weight-shifting activities, static standing balance, gait training with a walker	10 reps × 3 sets
Month 4	Partial weight-bearing ambulation, mini squats, standing hip strengthening, heel raises (as tolerated), step-up exercises, stationary cycling without resistance (10 minutes), static and dynamic balance exercises	10 reps × 3 sets
Month 5	Progressive resisted exercises, sit-to-stand progression, weight-shifting drills, tandem standing, step-up and step-down exercises, walking endurance training	10 reps × 3 sets
Month 6	Full weight-bearing training (as permitted), stair climbing practice, stationary cycling with mild resistance, dynamic balance training, functional strengthening exercises, community ambulation training	10 reps × 3 sets
Month 7	Single-leg stance progression, balance board training, resistance band strengthening, functional reaching activities, and advanced gait training	10 reps × 3 sets
Month 8	Treadmill walking, lunges (within tolerance), functional task training, endurance walking programme, proprioceptive training	20 minutes
Month 9	Squat progression, step training, functional lifting activities, theraband progression, and outdoor ambulation on uneven surfaces	10 reps × 3 sets
Month 10	Advanced strengthening exercises, multidirectional stepping, obstacle negotiation training, functional mobility drills, aerobic conditioning	10 reps × 3 sets
Month 11	Advanced balance exercises, community mobility training, functional independence training, endurance progression, home exercise progression	10 reps × 3 sets
Month 12	Independent strengthening programme, stretching exercises, functional activity practice, outdoor walking programme, maintenance exercise programme	10 reps × 3 sets

Result

The patient's pain intensity was assessed using the Numeric Pain Rating Scale (NPRS). Pain intensity in this case was evaluated using the NPRS. While the NPRS is a subjective patient-reported outcome measure and may be influenced by psychological and individual factors, it remains one of the most widely accepted clinical tools for assessing pain because of its ease of administration, good test-retest reliability, and responsiveness to changes following rehabilitation. To reduce reliance on a single subjective measure, pain scores were interpreted alongside objective clinical outcomes, including range of motion, muscle strength, gait performance, and functional outcome measures. A reduction in pain was observed following the intervention. Pain at rest decreased from 5/10 before treatment to 3/10 after treatment, while pain during movement decreased from 8/10 to 6/10. These findings indicate an improvement in pain levels, suggesting that the combined physiotherapy rehabilitation and mindfulness-based intervention contributed to better pain management during both rest and functional activities, as shown in Table 2.

**Table 2:** Numerical Pain Rating Scale (NPRS)

NPRS	Pre-intervention	Post-intervention
At rest	5/10	3/10
On activity	8/10	6/10

Manual Muscle Testing demonstrated improvements in the strength of the affected lower limb muscles following the intervention. Right hip flexor and extensor strength improved from 3/5 to 4/5, knee flexor and extensor strength improved from 2/5 to 3/5, and ankle plantar flexor and dorsiflexor strength improved from 1/5 to 3/5. These findings indicate progressive restoration of lower limb muscle strength, contributing to enhanced functional mobility and weight-bearing capacity, as shown in Table 3.

**Table 3:** Manual Muscle Testing (MMT)

MMT	Pre-intervention		Post-intervention	
	Right	Left	Right	Left
Hip flexors	3/5	4/5	4/5	4/5
Hip extensors	3/5	4/5	4/5	4/5
Knee flexors	2/5	4/5	3/5	4/5
Knee extensors	2/5	4/5	3/5	4/5
Ankle plantar flexors	1/5	4/5	3/5	4/5
Ankle dorsi flexors	1/5	4/5	3/5	4/5

Post-intervention assessment demonstrated notable improvements in the range of motion of the affected right lower limb. Right hip flexion improved from 0-70° to 0-90°, hip extension from 0-10° to 0-15°, knee flexion from 0-30° to 0-90°, plantar flexion from 0-10° to 0-15°, and dorsiflexion from 0-10° to 0-15°. These improvements indicate enhanced joint mobility and functional movement capacity, contributing to better performance of daily activities and progression toward independent ambulation, as shown in Table 4.

**Table 4:** Range of motion ROM: range of motion

ROM	Pre-intervention		Post-intervention	
	Right	Left	Right	Left
Hip flexion	0-70	0-100	0-90	0-110
Hip extension	0-10	0-20	0-15	0-20
Knee flexion	0-30	0-100	0-90	0-110
Knee extension	30-0	100-0	90-0	110-0
Plantar flexion	0-10	0-20	0-15	0-25
Dorsi flexion	0-10	0-20	0-15	0-25

The patient's Mindful Attention Awareness Scale (MAAS) score [13] was improved from 40/90 before the intervention to 60/90 after the intervention, indicating enhanced mindfulness, increased present-moment awareness, and improved psychological well-being following the combined physiotherapy and mindfulness-based rehabilitation program, as shown in Table 5.

**Table 5:** Mindful Attention Awareness Scale (MAAS)

MAAS	
Pre-intervention	40/90
Post-intervention	60/90

The patient's Barthel Index score [14] improved from 30/100 pre-intervention to 70/100 post-intervention, demonstrating substantial gains in functional independence and the ability to perform daily activities. This improvement reflects the positive impact of the rehabilitation program on overall functional capacity and quality of life, as shown in Table 6.

**Table 6:** Barthel index

Barthel index	
Pre-intervention	30/100
Post-intervention	70/100

## Discussion

The use of the Ilizarov fixator in tibial fractures resulted in high percentages of effective bone healing, even in complicated fractures with delayed union. They stated that the technique not only corrected the abnormalities caused by the fractures but also allowed for the restoration of limb length and function [15]. Borzunov et al. reported cases of delayed and non-union fractures treated with the Ilizarov fixator, and it was noted that, while the technique is effective in promoting bone regeneration and healing, managing fractures of delayed union requires careful attention to patient-specific factors, such as bone quality, infection presence, and bone alignment [16]. Mundada et al. concluded that a multidisciplinary rehabilitation that includes a specific surgical procedure and an individualised physical therapy management protocol for CO managed with sequestrectomy and fixation with an Ilizarov ring results in improved functional ability and thus plays an important role in quick and effective rehabilitation [17].

Veehof et al. studied that emotional factors can interfere with the rehabilitation process and reduce the overall effectiveness of physical therapy [18]. Impieri et al. studied that, in the case of long-standing fractures, such as a proximal tibia fracture, the psychological impact of immobilization for a long period of time and physical limitation can be significant. The young age patient having a delayed or non-union tibia fracture often experiences frustration, stress, and anxiety due to prolong healing process and extended rehabilitation time [19].

The findings of the present study are consistent with previous literature demonstrating the effectiveness of the Ilizarov fixator in the management of complex tibial fractures. Wani et al. reported that the early application of the Ilizarov ring fixator in type II, IIIA, and IIIB open tibial shaft fractures resulted in satisfactory fracture union, reduced infection rates, and favorable functional outcomes [20]. Similarly, Cibura et al. highlighted the versatility of the Ilizarov technique in treating both open and closed tibial shaft fractures, particularly in patients with complex clinical presentations. The circular external fixation system provides stable fracture fixation while allowing controlled weight-bearing and preservation of soft tissues, which are crucial factors for successful fracture healing. These findings support the use of the Ilizarov method as a reliable treatment option for complicated tibial fractures requiring prolonged stabilization and deformity correction [21].

Furthermore, rehabilitation plays a vital role in optimizing functional recovery following Ilizarov fixation. Pathan et al. demonstrated that a comprehensive physiotherapeutic program significantly improved joint mobility, muscle strength, gait pattern, and overall functional independence in a patient with a proximal tibial fracture managed with an Ilizarov fixator and associated foot drop [22]. In addition, Mohib et al. reported successful bone regeneration and limb reconstruction using tetrafocal bone lengthening with the Ilizarov frame in a patient with segmental tibial bone loss, emphasizing the device's capacity for managing extensive bone defects. Collectively, these studies suggest that the Ilizarov fixator, when combined with structured physiotherapy, can facilitate fracture union, enhance functional outcomes, and restore mobility in patients with complex tibial injuries. The present findings further reinforce the importance of integrating surgical stabilization with targeted rehabilitation strategies to achieve optimal patient recovery [23].

Our study presented a case of an infective eight-month-old non-united proximal tibia fracture, previously managed with open reduction and internal fixation, along with a wound on the proximal tibia. They underwent an operation consisting of fasciotomy and an external fixator. As the previous fixation was infected and the wound developed, it was replaced by the Ilizarov ring fixator. We started physiotherapy management postoperatively on day two. In order to prevent and manage immobility-related difficulties, the case's complexity required thorough assessment and timely postoperative care. Since physiotherapy is essential to patients' postoperative rehabilitation, it should be promoted from the initial stages of the healing process in order to speed up recovery through early mobilisation and encourage early discharge.

Following a 12-month rehabilitation program consisting of structured physiotherapy with adjunctive mindfulness therapy, the patient demonstrated improvements in pain, functional mobility, and quality of life. As this is a single case report, the independent contribution of mindfulness therapy cannot be determined, and the observed improvements likely reflect the combined effects of multidisciplinary rehabilitation, surgical management, and the natural healing process. The patient demonstrated marked improvements in pain, muscle strength, joint mobility, mindfulness, and functional independence. NPRS scores decreased from 5/10 to 3/10 at rest and from 8/10 to 6/10 during movement, indicating effective pain management. Manual Muscle Testing revealed improvements in the strength of the affected lower-limb muscles, with right hip flexors and extensors improving from 3/5 to 4/5, knee flexors and extensors from 2/5 to 3/5, and ankle plantar flexors and dorsiflexors from 1/5 to 3/5. Range of motion also improved substantially, with right hip flexion increasing from 70° to 90°, hip extension from 10° to 15°, knee flexion from 30° to 90°, plantar flexion from 10° to 15°, and dorsiflexion from 10° to 15°. Furthermore, the MAAS score improved from 40/90 to 60/90, reflecting enhanced mindfulness and psychological well-being, while the Barthel index increased from 30/100 to 70/100, indicating greater independence in activities of daily living.

These improvements can be attributed to the comprehensive rehabilitation approach that emphasized early mobilization, progressive strengthening, range-of-motion exercises, balance training, gait re-education, and functional task practice. Physiotherapy played a crucial role in minimizing the adverse effects of prolonged immobilization associated with Ilizarov fixation, such as muscle weakness, joint stiffness, and functional limitations. The findings are consistent with previous studies reporting that structured physiotherapy following Ilizarov fixation promotes restoration of limb function, improves mobility, and facilitates return to daily activities. Additionally, the incorporation of mindfulness training may have contributed to reduced pain perception, improved emotional well-being, and better engagement in rehabilitation. Overall, the combined physiotherapy and mindfulness-based intervention was associated with meaningful improvements in both physical and psychological outcomes, supporting its role in the management of complex tibial fractures treated with Ilizarov fixation.

Therefore, adjuvant therapy, such as mindfulness-based intervention, should be administered to address the psychological and emotional aspects. Mindfulness therapy, which includes meditation, breathing exercises, and body awareness, has been demonstrated to reduce pain and stress and improve mood, which helps improve the overall quality of life and reduce the rehabilitation timeline following complex fractures. While in the above case study, mindfulness therapy was used along with physical rehabilitation, which helped the patient improve the postoperative psychological outcomes. As the psychological outcomes improved, it helped reduce pain and overall functional ability throughout the rehabilitation period.

## Conclusions

This case highlights the role of a structured physiotherapy rehabilitation programme in improving pain, mobility, muscle strength, gait, and functional independence in a patient with an infected proximal tibial fracture managed with an Ilizarov external fixator. Mindfulness therapy was included as an adjunctive intervention to support psychological well-being during prolonged rehabilitation. However, as this is a single case report, the independent effect of mindfulness therapy cannot be established. Further controlled studies are needed to evaluate its additional benefits in orthopaedic rehabilitation.

## References

[1] Somaiya K, Samal S, Boob M (2023). Case report: effectiveness of physiotherapy rehabilitation in a patient with compound grade IIIC proximal third tibia fracture along with vascular injury. F1000Research.

[2] Miller NC, Askew AE (2007). Tibia fractures: an overview of evaluation and treatment. Orthop Nurs.

[3] Wennergren D, Bergdahl C, Ekelund J, Juto H, Sundfeldt M, Möller M (2018). Epidemiology and incidence of tibia fractures in the Swedish Fracture Register. Injury.

[4] Kim PH, Leopold SS (2012). Gustilo-Anderson classification. Clin Orthop Relat Res.

[5] Saeidi M, Barnes SC, Berthaume MA, Holthof SR, Milandri GS, Bull AM, Jeffers J (2022). Low-cost locally manufacturable unilateral imperial external fixator for low- and middle-income countries. Front Med Technol.

[6] Dhaniwala N, Jadhav S, Chirayath A, Saoji A (2023). Ilizarov ring fixator in the lower limb for 2000 days: a case report. Cureus.

[7] Malkova TA, Borzunov DY (2021). International recognition of the Ilizarov bone reconstruction techniques: current practice and research (dedicated to 100th birthday of G. A. Ilizarov). World J Orthop.

[8] Reynolds A, Hamidian Jahromi A (2022). Improving postoperative care through mindfulness-based and isometric exercise training interventions: systematic review. JMIR Perioper Med.

[9] Desbordes G, Gard T, Hoge EA (2015). Moving beyond mindfulness: defining equanimity as an outcome measure in meditation and contemplative research. Mindfulness (N Y).

[10] Creswell JD (2017). Mindfulness interventions. Annu Rev Psychol.

[11] Bagheri S, Naderi A, Mirali S, Calmeiro L, Brewer BW (2021). Adding mindfulness practice to exercise therapy for female recreational runners with patellofemoral pain: a randomized controlled trial. J Athl Train.

[12] Chen X, Tian C, Zhang Y, Fu Y, Han W, Zhang R (2025). The role of mindfulness decompression therapy in managing acute stress disorder in traumatic fracture patients. Actas Esp Psiquiatr.

[13] Brown KW, Ryan RM (2003). The benefits of being present: mindfulness and its role in psychological well-being. J Pers Soc Psychol.

[14] Mahoney FI, Barthel DW (1965). Functional evaluation: the Barthel index. Md State Med J.

[15] Messner J, Johnson L, Taylor DM, Harwood P, Britten S, Foster P (2018). Treatment and functional outcomes of complex tibial fractures in children and adolescents using the Ilizarov method. Bone Joint J.

[16] Borzunov DY, Kolchin SN, Malkova TA (2020). Role of the Ilizarov non-free bone plasty in the management of long bone defects and nonunion: problems solved and unsolved. World J Orthop.

[17] Mundada PH, Patil DS (2022). Early physiotherapy as an adjunct to surgical approach in case of chronic tibial osteomyelitis treated with sequestrectomy and an Ilizarov ring fixator in a 14-year-old schoolgirl: a case report. Cureus.

[18] Veehof MM, Oskam MJ, Schreurs KM, Bohlmeijer ET (2011). Acceptance-based interventions for the treatment of chronic pain: a systematic review and meta-analysis. Pain.

[19] Impieri L, Mazzoli G, Pezzi A, Peretti GM, Mangiavini L, Rossi N (2025). Mindfulness-based intervention for non-pharmacological pain control after total hip and total knee arthroplasty: a systematic review and meta-analysis of randomized controlled trials. J Orthop Rep.

[20] Wani N, Baba A, Kangoo K, Mir M (2011). Role of early Ilizarov ring fixator in the definitive management of type II, IIIA and IIIB open tibial shaft fractures. Int Orthop.

[21] Cibura C, Ull C, Rosteius T (2024). The use of the Ilizarov fixator for the treatment of open and closed tibial shaft and distal tibial fractures in patients with complex cases. Z Orthop Unfall.

[22] Pathan AF, Samal S (2024). A comprehensive physiotherapeutic intervention for complex proximal tibia fracture with Ilizarov fixator and foot drop in 18-year-old adult: a case report. Cureus.

[23] Mohib Y, Ali U, Mariam F, Slote MB, Ali Z, Rashid HU (2025). Tetrafocal bone lengthening by Ilizarov frame application in a patient with segmental tibial bone loss: a case report. JBJS Case Connect.

